# UIF, a New mRNA Export Adaptor that Works Together with REF/ALY, Requires FACT for Recruitment to mRNA

**DOI:** 10.1016/j.cub.2009.09.041

**Published:** 2009-12-01

**Authors:** Guillaume M. Hautbergue, Ming-Lung Hung, Matthew J. Walsh, Ambrosius P.L. Snijders, Chung-Te Chang, Rachel Jones, Chris P. Ponting, Mark J. Dickman, Stuart A. Wilson

**Affiliations:** 1 Department of Molecular Biology and Biotechnology, The University of Sheffield, Firth Court, Western Bank, Sheffield S10 2TN, UK; 2 Department of Chemical and Process Engineering, The University of Sheffield, The Sheffield Bioincubator, 40 Leavygreave Road, Sheffield S3 7RD, UK; 3 Department of Physiology, Anatomy and Genetics, MRC Functional Genomics Unit, University of Oxford, Le Gros Clark Building, Oxford OX1 3QX, UK

**Keywords:** RNA

## Abstract

Messenger RNA (mRNA) export adaptors play an important role in the transport of mRNA from the nucleus to the cytoplasm. They couple early mRNA processing events such as 5′ capping and 3′ end formation with loading of the TAP/NXF1 export receptor onto mRNA. The canonical adaptor REF/ALY/Yra1 is recruited to mRNA via UAP56 and subsequently delivers the mRNA to NXF1 [1]. Knockdown of UAP56 [2, 3] and NXF1 [4–7] in higher eukaryotes efficiently blocks mRNA export, whereas knockdown of REF only causes a modest reduction, suggesting the existence of additional adaptors [8–10]. Here we identify a new UAP56-interacting factor, UIF, which functions as an export adaptor, binding NXF1 and delivering mRNA to the nuclear pore. REF and UIF are simultaneously found on the same mRNA molecules, and both proteins are required for efficient export of mRNA. We show that the histone chaperone FACT specifically binds UIF, but not REF, via the SSRP1 subunit, and this interaction is required for recruitment of UIF to mRNA. Together the results indicate that REF and UIF represent key human adaptors for the export of cellular mRNAs via the UAP56-NXF1 pathway.

## Results and Discussion

### UIF Is a Nuclear RNA-Binding Protein with a UAP56-Binding Motif

**Figure 1:**
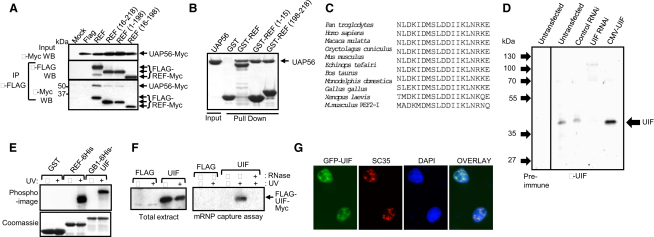
Identification and Characterization of UIF (A) Western analysis of coimmunoprecipitation (CoIP) between FLAG-REF2-I fusions and UAP56-Myc expressed in 293T cells. Immunoprecipitations used the FLAG antibody, and proteins were detected with both FLAG and Myc antibodies. (B) GST, GST-REF, and GST-REF peptides were used in pull-down assays with recombinant UAP56. Proteins were detected via Coomassie staining. (C) Alignment of the N-terminal UAP56-binding motif from REF2-I with vertebrate UIF genes. (D) Western blot analysis of 293T cell extracts with an antibody raised against recombinant UIF. Extracts from 293T cells transfected with an untagged cDNA for UIF (CMV-UIF), a control RNA interference (RNAi) vector (control RNAi), or a vector expressing a microRNA (miRNA) targeting UIF (UIF RNAi) were used with the UIF antisera (right). An untransfected 293T cell extract was also probed with a preimmune serum (left). (E) UV crosslinking of ^32^P-labeled RNA with recombinant REF2-I and UIF. Proteins were resolved by SDS-PAGE and RNA detected by phosphoimaging (top), and proteins were detected by Coomassie staining (bottom). (F) Messenger ribonucleoprotein (mRNP) capture assay with extract from 293T cells transfected with FLAG-UIF. The FLAG-UIF was detected via western blotting with the FLAG antibody. The left panel shows the input extracts for the mRNP capture assay; the right panel shows the results of the mRNP capture assay. (G) Localization of GFP-UIF in COS-7 cells. Cells were stained with an antibody to SC35 and DAPI.

To identify export factors with functions possibly overlapping those of REF, we examined its interaction with UAP56. A C-terminal peptide from REF is involved in binding UAP56 [11], and an N-terminal peptide from REF is involved in binding a UAP56 paralog, DDX39 [12]. The relative contribution of each peptide to UAP56 binding was assessed via a coimmunoprecipitation (CoIP) assay (Figure 1A). Although loss of either peptide (amino acids 1–15 or 198–218) decreased binding to UAP56, loss of both reduced the interaction to background levels, suggesting that both peptides contribute to the interaction between REF and UAP56. The ability of the N- and C-terminal REF peptides to bind UAP56 was further assessed with the isolated peptides individually fused to GST in pull-down assays (Figure 1B). Both peptides bound UAP56, thus defining two distinct UAP56-binding motifs. A basic local alignment search tool (BLAST) database search with the N-terminal REF peptide identified an uncharacterized protein that we named UIF for UAP56-interacting factor (Figure 1C). The UAP56-binding motif was the only region within UIF with homology to REF. UIF is conserved in vertebrates (Figure 1C) and is found in other animals and plants (see Figure S1 available online), although it was not readily identifiable among *C. elegans* and *Drosophila* proteins.

By using an antiserum raised to UIF, we detected it in extracts from 293T cells as a 37 kDa protein (Figure 1D). The levels of UIF were increased when cells were transfected with a UIF cDNA expression vector and reduced when cells expressed a microRNA (miRNA) targeting UIF messenger RNA (mRNA), indicating that the antibody recognized UIF. We examined expression of UIF in chick embryos and found a widespread expression pattern during development (Figure S2). The expression pattern was similar to that observed for REF and SF2/ASF. SF2/ASF functions in many aspects of mRNA metabolism, including splicing, translation, and mRNA stability [13], but also binds NXF1 and functions as an mRNA export adaptor [14, 15]. Quantitative reverse transcriptase-polymerase chain reaction (qRT-PCR) analysis revealed expression of UIF mRNA in all cell lines tested (Figure S3). As an mRNA export factor, UIF is predicted to bind RNA, yet its amino acid sequence contains no recognizable RNA-binding motifs. Therefore, we carried out UV crosslinking assays with recombinant REF and UIF and found that both proteins crosslinked with RNA efficiently (Figure 1E). Furthermore, UIF was found associated with poly(A)^+^ RNA in vivo via a messenger ribonucleoprotein (mRNP) capture assay (Figure 1F). A GFP-UIF fusion was localized in the nucleoplasm with some accumulation in nuclear speckles (Figure 1G), together with SC35, in common with other mRNA export factors [7, 16]. The nuclear localization and ability to bind RNA are consistent with a role for UIF in mRNA processing and/or export.

### Mutually Exclusive Binding of UAP56 and NXF1 to UIF

**Figure 2:**
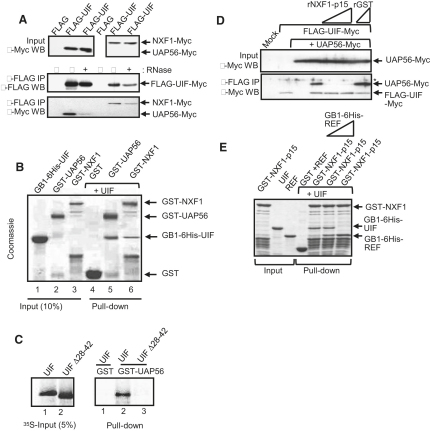
UIF Binds UAP56 and NXF1 (A) Western analysis of CoIP between FLAG-UIF and Myc-tagged NXF1 and UAP56. Immunoprecipitations were carried out with FLAG antibody and proteins detected with both FLAG and Myc antibodies. (B) Pull-down assays with the indicated GST fusion proteins and purified UIF in the presence of RNase. Proteins were detected by Coomassie staining. (C) Pull-down of GST-UAP56 with the indicated ^35^S-labeled UIF proteins generated with rabbit reticulocyte lysate. (D) Western analysis of CoIP between FLAG-UIF and Myc UAP56 in the presence of recombinant NXF1-p15. The asterisk marks a nonspecific band. The FLAG antibody was used for immunoprecipitation, and proteins were detected with the 9E10 Myc antibody. (E) Pull-down competition assay with GST-NXF1-p15 complexed with GB1-UIF and increasing amounts of GB1-6His-REF2-I. Proteins were detected via Coomassie staining.

The ability of UIF to bind both UAP56 and NXF1 was investigated via CoIP assays (Figure 2A). While UIF bound both proteins, interactions were reduced in the presence of RNase but were still detectable above background levels. UIF interactions with UAP56 and NXF1 are direct because they occur with recombinant proteins in the presence of RNase (Figure 2B). Furthermore, an internal deletion of the UAP56-binding motif within UIF prevented its interaction with GST-UAP56 in pull-down assays (Figure 2C).

To examine whether UIF binds UAP56 and NXF1-p15 simultaneously, we coimmunoprecipitated UIF with UAP56 in the presence of increasing amounts of NXF1-p15 (Figure 2D). NXF1-p15 efficiently displaced UAP56 from UIF, whereas GST did not. This indicates that UAP56 and NXF1 bind UIF in a mutually exclusive manner. Similarly, UAP56 and NXF1 binding to REF are mutually exclusive [17]. NXF1 binds REF and the coadaptor THOC5 simultaneously [10]; thus, to clarify whether UIF might be a coadaptor, we examined its ability to bind NXF1 in the presence of REF (Figure 2E). REF displaced UIF bound to NXF1, thus, UIF and REF binding to NXF1 are mutually exclusive. These data indicate that UIF represents a new mRNA export adaptor rather than coadaptor.

### UIF Is Required for Efficient Bulk mRNA Export

**Figure 3:**
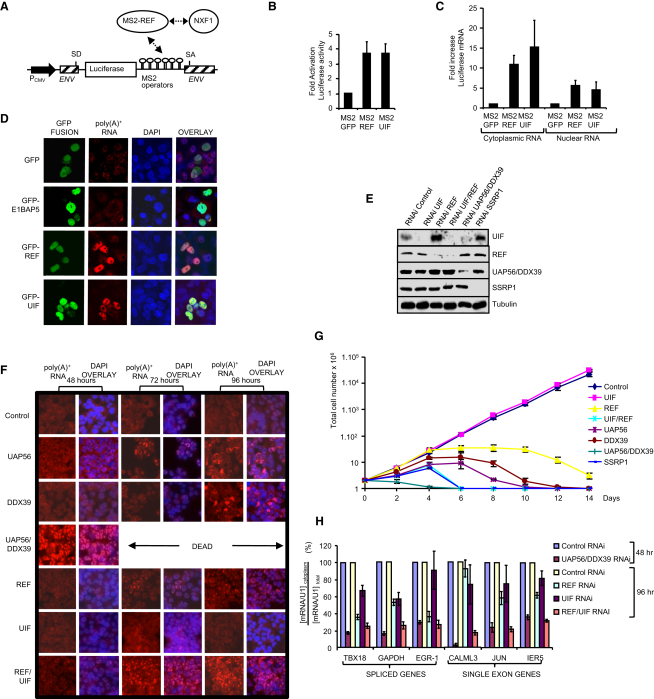
UIF and REF Are Required for Bulk Poly(A)^+^ RNA Export (A) Schematic of the mRNA export reporter vector. The splice donor (SD) and acceptor (SA) sites are indicated. (B and C) Tethering of REF and UIF to the reporter RNA triggers luciferase expression (B) and increases cytoplasmic levels of the reporter RNA, assayed by reverse transcriptase-polymerase chain reaction (qRT-PCR) (C). Error bars represent the standard error of the mean for three or more experiments. (D) Overexpression of GFP-REF and GFP-UIF in COS-7 cells leads to nuclear accumulation of poly(A)^+^ RNA. Panels are shown at the same exposure. Poly(A)^+^ RNA was detected via fluorescence in situ hybridization (FISH) with a Cy3-oligo-dT probe. (E) Western analysis of stable cell lines following tetracycline-induced expression of miRNAs for mRNA export factors. Proteins were detected with antibodies to the endogenous proteins indicated on the right of each panel. (F) Localization of poly(A)^+^ RNA following induction of miRNAs targeting export factors for 48, 72, or 96 hr. UAP56/DDX39 knockdown leads to cell death by 72 hr. All equivalent panels are shown at the same exposure. (G) Growth of stable cell lines following induction of miRNAs targeting the indicated genes. Error bars represent the standard deviation based on three independent experiments. (H) Quantitative RT-PCR analysis of the RNA distribution in stable 293 cells expressing miRNAs to the indicated genes. The ratio of cytoplasmic to total RNA normalized to U1 small nuclear RNA (snRNA) levels is displayed at 48 hr and 96 hr with values for control samples set at 100%. Error bars represent standard error of the mean for three or more experiments.

To address the role of UIF in mRNA export in vivo, we investigated its ability to function in a tethered export assay [18]. The assay involves a luciferase gene within an inefficiently spliced intron, together with bacteriophage MS2 RNA operator sites (Figure 3A). The unspliced pre-mRNA is normally retained in the nucleus. However, an MS2 coat protein-mRNA export factor fusion, when bound to the MS2 operators, overcomes nuclear retention, leading to export of the pre-mRNA and luciferase expression. Tethering of both REF and UIF led to increased luciferase activity and increased cytoplasmic levels of reporter pre-mRNA, indicating that UIF functions as an mRNA export factor (Figures 3B and 3C). Increased nuclear levels of reporter RNA were also seen in the presence of MS2-REF and MS2-UIF, which may reflect greater stability of reporter RNA destined for export, as observed previously for Gag-Pol mRNA in the presence of Rev [19]. The levels of the yeast ortholog of REF (Yra1p) are critical for efficient mRNA export, and overexpression of Yra1p blocks export [20]. Similarly, REF and UIF overexpression caused nuclear accumulation of poly(A)^+^ RNA, whereas GFP and another NXF1-binding protein, E1B-AP5, did not (Figure 3D). We conclude that abnormally high cellular levels of REF and UIF impair mRNA export.

To assess the relative contribution of REF and UIF to mRNA export, we generated 293 cell lines that could be induced to express miRNAs targeting specific export factors. Knockdown of target mRNAs was confirmed by qRT-PCR analysis. This analysis also revealed that double knockdown of REF and UIF led to a large increase in UAP56 and DDX39 mRNA levels (Figure S4A). This effect was specific for UAP56/DDX39, and none of the seven genes tested that were unrelated to mRNA export factors showed a significant increase in mRNA levels following REF/UIF knockdown, although Jun mRNA showed an increase in levels following UAP56/DDX39 knockdown (Figure S4B). The increased transcription of UAP56/DDX39 observed when REF/UIF levels are reduced is not unprecedented. Earlier work with *Drosophila* S2 cells has shown that when NXF1, p15, or UAP56 is individually knocked down, this leads to compensatory increases in the mRNA levels for the other two export factors together with increases in REF1 mRNA levels, although this does not lead to increased protein levels. Thus, a feedback loop exists whereby a block in mRNA export leads to upregulation of mRNA export factor genes [6]. The results presented here extend that feedback loop to include UIF. Interestingly, western analysis of the cell lines revealed that knockdown of REF led to a concomitant increase in UIF protein levels (Figure 3D), indicating that the relative amounts of the two proteins are coupled in vivo. In contrast, UAP56/DDX39 protein levels did not alter significantly following knockdown of REF/UIF, despite the large increase in mRNA levels, consistent with earlier observations [6]. The increase in UIF levels following REF knockdown probably represents a survival mechanism whereby the cell tries to maintain normal mRNA export by increasing the levels of the alternative export adaptor. This may also account for the observation that REF knockdown leads to modest reductions in mRNA export.

The effects of export factor depletion on poly(A)^+^ RNA export were examined via oligo-dT fluorescence in situ hybridization (FISH) following induction of miRNA expression (Figure 3F). Single knockdowns of UAP56 or DDX39 led to nuclear accumulation of poly(A)^+^ RNA, which was most apparent at 96 hr. In contrast, knockdown of UAP56/DDX39 led to robust nuclear accumulation of poly(A)^+^ RNA as early as 48 hr, as shown previously [21], and cells were dead by 72 hr. Cells depleted of REF showed a modest accumulation of poly(A)^+^ RNA as reported previously [10], which was apparent in cells at 72 hr and more pronounced at 96 hr. However, the 96 hr phenotype was far less severe than that observed following UAP56/DDX39 knockdown. Knockdown of UIF gave no phenotype readily detectable by FISH at either 96 hr (Figure 3F) or 8 days (data not shown). In contrast, knockdown of REF/UIF showed a robust nuclear accumulation of poly(A)^+^ RNA, clearly visible in the majority of cells as early as 72 hr. At 96 hr, the phenotype was almost as severe as that observed with UAP56/DDX39 knockdown, indicating that both REF and UIF are essential for efficient mRNA export.

The UIF knockdown did not affect cell growth, and knockdown of REF impaired cell growth but did not kill all cells within 14 days. In contrast, double knockdown of REF/UIF killed cells within 6 days (Figure 3G), consistent with the robust nuclear accumulation of poly(A)^+^ RNA within 4 days (Figure 3F). Growth curves also confirmed that UAP56/DDX39 knockdown had the most profound effect, killing cells within 4 days, showing a much stronger effect than single UAP56 or DDX39 knockdowns. In contrast, the cell lines grew well when miRNA expression was not induced (Figure S5). To further analyze the effects on mRNA export, we performed qRT-PCR analysis on total and cytoplasmic mRNA levels for both spliced and unspliced genes (Figure 3H). REF knockdown reduced cytoplasmic mRNA levels for 5 out of 6 genes tested, with both spliced and unspliced genes affected. Reductions in cytoplasmic RNA levels were more modest following UIF knockdown for most genes, though GAPDH mRNA export was reduced by ∼40%, a level similar to the reduction seen following REF knockdown. However, REF/UIF knockdown led to a further drastic reduction in cytoplasmic mRNA levels, similar to those seen with UAP56/DDX39 knockdown. This suggests that REF and UIF represent the two major adaptors used for export by the mRNAs tested. The onset of the export block following REF/UIF knockdown is delayed compared with the effects following UAP56/DDX39 depletion. This may be caused by the additional roles that UAP56 and DDX39 play in splicing and 3′ end processing [22–24], because perturbation of these processes will also impact on mRNA export.

### UIF Associates with the FACT Histone Chaperone via SSRP1

**Figure 4:**
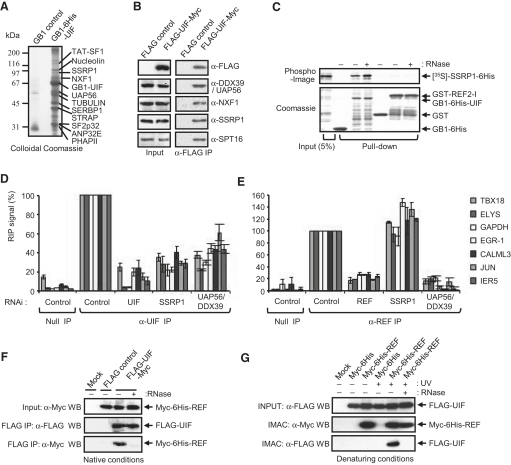
SSRP1 Is Required to Load UIF onto mRNA (A) Proteins purified by a control GB1 tag or via GB1-6His-UIF affinity chromatography were separated by SDS-PAGE. The indicated proteins were identified by mass spectrometry. (B) Western analysis of FLAG-UIF immunoprecipitates probed with the indicated antibodies was used to confirm interactions. (C) Pull-down assays with UIF and REF fusions and radiolabeled SSRP1 generated in reticulocyte lysate. (D and E) qRT-PCR analysis of mRNA, which coimmunoprecipitates with endogenous UIF (D) or REF (E) following knockdown of the indicated mRNA export factors. Values are expressed as a percentage relative to the value seen in the control RNAi immunoprecipitations. Error bars represent the standard error of the mean for three or more experiments. (F) CoIP of UIF and REF under native conditions. Proteins were immunoprecipitated with FLAG antibody. The following abbreviations are used: IP, immunoprecipitation; WB, western blot. (G) Copurification of UIF and REF under denaturing conditions via immobilized metal affinity chromatography (IMAC).

GB1-UIF fusion affinity chromatography was used to identify UIF-interacting proteins by mass spectrometry (Figure 4A). This analysis identified NXF1 and UAP56 together with other proteins. Attention was focused on SSRP1, which was previously implicated in mRNA export [6, 25]. SSRP1 and SPT16 form the FACT heterodimer, which functions as a histone chaperone and facilitates transcription by RNA polymerase II through chromatin [26]. UIF was found associated with both subunits of FACT by CoIP (Figure 4B), and the UIF:SSRP1 interaction was detectable with recombinant proteins (Figure S6A), demonstrating that it is direct. By contrast, REF failed to interact with SSRP1 in vitro (Figure 4C) and in CoIP assays (Figure S6B). The knockdown of SSRP1 (Figure 3D) led to a significant increase in UIF levels in vivo, indicating that levels of these two proteins are coupled. Although SSRP1 knockdown gives a partial mRNA export block in S2 cells [6, 25], no such phenotype was observed in 293 cells via FISH before they died, consistent with the lack of phenotype observed following UIF knockdown. We explored whether UIF bound other proteins involved in mRNA export via CoIP assays (Figure S6B). UIF was found associated with the nuclear CAP-binding complex subunit CBP80 in common with REF [27] and interacts with components of TREX [28].

### SSRP1 and UAP56 Are Required for UIF Recruitment to mRNA

Because FACT associates with RNA polymerase II [29], it is well placed to contribute to loading UIF onto nascent mRNA. To investigate this, we carried out RNA immunoprecipitation analysis. UIF coimmunoprecipitated with all spliced and unspliced single-exon mRNAs tested (Figure 4D). When UIF levels were reduced by RNA interference, the amount of recovered mRNA dropped dramatically, indicating that the UIF antibody was specifically immunoprecipitating UIF in control samples. Following SSRP1 knockdown, levels of UIF associated with mRNAs were also reduced, despite input levels of mRNA remaining similar (Figure S8). Therefore, SSRP1 is required for efficient loading of UIF onto mRNA in vivo. In contrast, SSRP1 knockdown had no effect on the ability of REF to interact with the same mRNAs in vivo (Figure 4E). Interestingly, another histone H3 chaperone, Spt6, also plays a role in mRNA export, binding Iws1, which in turn triggers recruitment of REF to Spt6-responsive genes [30, 31]. We further analyzed whether UIF required UAP56/DDX39 for efficient loading on mRNA (Figures 4D and 4E). The amount of REF associated with the mRNAs tested was typically reduced by ∼80%–90% following UAP56/DDX39 knockdown. In contrast, UIF association with the mRNAs tested was only reduced by ∼50%–60% following UAP56/DDX39 knockdown. Thus, REF loading onto mRNA is critically dependent on UAP56/DDX39, whereas UIF is less so. The residual loading of UIF onto mRNA in the absence of UAP56/DDX39 may result from the involvement of SSRP1 in recruitment of UIF to mRNA. SSRP1 was first implicated in mRNA export through studies in *Drosophila* S2 cells, where it was observed that SSRP1 knockdown led to a partial mRNA export block [6], yet surprisingly we have been unable to identify a UIF ortholog in *Drosophila*. Furthermore, the knockdown of REF proteins in *Drosophila* does not block mRNA export [8], indicating that this organism possesses alternative adaptor proteins that may well utilize SSRP1.

### REF and UIF Are Simultaneously Found on the Same mRNA Molecules

The RNA immunoprecipitation showed that REF and UIF were associated with the same mRNAs. In fact, REF and UIF coimmunoprecipitate (Figure 4F), but this interaction is sensitive to ribonuclease, suggesting that the CoIP is caused by mRNA bridging the interaction. To exclude the possibility that the CoIP was in part due to a protein-protein interaction stimulated by the presence of RNA, we crosslinked proteins to mRNA in vivo and purified them under denaturing conditions. Copurification of REF and UIF was still observed, but only following RNA crosslinking and omission of ribonuclease (Figure 4G). We conclude that REF and UIF simultaneously bind the same mRNA molecule in vivo.

In conclusion, we have identified a novel mRNA export adaptor that utilizes the UAP56/NXF1 pathway. UIF is loaded onto mRNA via FACT and, together with REF, it ensures efficient mRNA export. UIF and REF are simultaneously associated with all mRNAs tested, irrespective of whether they are spliced and their combined loss leads to a dramatic nuclear accumulation of mRNA. Therefore, despite the identification of other mRNA export adaptors such as SR proteins [14] that bind NXF1, these proteins seem unable to maintain export of the mRNAs that we have tested in the absence of REF and UIF. The reliance on at least two adaptors for efficient mRNA export, which in turn may recruit two NXF1 molecules, probably ensures efficient translocation of the mRNA across the nuclear pore by providing multiple export signals. This situation is reminiscent of export of ribosomal subunits that utilize multiple export receptors [32]. With the identification of UIF, the repertoire of mRNA export adaptors is expanded and now provides a clear framework to explore the extent of transcript-specific mRNA export in human cells.

## Supplemental Data

Document S1. Supplemental Experimental Procedures, One Table, and Eight Figures
