# Supplementary material for: UIF, a New mRNA Export Adaptor that Works Together with REF/ALY, Requires FACT for Recruitment to mRNA
Source: Curr Biol. 2009 Dec 1;19(22):1918–24. doi: 10.1016/j.cub.2009.09.041 (PMC2828547; doi:10.1016/j.cub.2009.09.041)
Supplement: Document S1. Supplemental Experimental Procedures, One Table, and Eight Figures [file mmc1.pdf]

Current Biology, Volume *19*

Supplemental Data

UIF, a New mRNA Export Adaptor that Works Together with REF/ALY, Requires FACT for Recruitment to mRNA

Guillaume M. Hautbergue, Ming-Lung Hung, Matthew J. Walsh, Ambrosius P.L. Snijders, Chung-Te Chang, Rachel Jones, Chris P. Ponting, Mark J. Dickman, and Stuart A. Wilson

Supplemental Experimental Procedures

Sequence Alignments

Multiple sequence alignment of animal and plant UIF-like sequences were constructed automatically using BLOCKMAKER [1] and prepared using CHROMA [2].

Plasmids, Antibodies, Cell Lines, and Transfections

Plasmids are listed in Table S1. UIF, UAP56/DDX39 and NXF1 antibodies were raised in rabbit (*Eurogentec*) using purified UIF-6His, DDX39-6His and NXF1 96-372-6His proteins expressed in *E. coli*. Mouse monoclonal THOC5 F6D antibody was a gift from A. Whetton and A.Pierce, Manchester. Purchased mouse antibodies include SSRP1 10D7, SPT16 8D2 (*Cambridge Biosciences*), ALY 11G5 (REF), tubulin B-5-1-2, FLAG M2 (*Sigma*) and MYC 9E10 (*Roche*). Human inducible RNAi cell line construction was as follows. Various miRNA hairpins were cloned using the BLOCK-IT PolII miR RNAi/meGFP kit (*Invitrogen*). Efficiency of RNAi hairpins was first assayed by Western blotting. Sequences targeted by successful miRNA hairpins were as follows: REF#5 (CCGATATTCAGGAACTCTTTG), UIF#5 (GTAACTCAGAAACCACGATTA), UAP56#2 (AGGAATATGAGCGCTTCTCTA), DDX39#3 (TCGTGCGGAATAGGAGCTTCA), SSRP1#2+5 (AGTGCATTACCTGTTCCTACA + TTTGCTCGTGGTACCACTACT). RNAi hairpins were then cloned as HindIII/NotI PCR fragments into pcDNA5-FRT/TO/His[3]. FLP-In T-REX 293 cells (*Invitrogen*) were transfected with a 6:4 ratio of pPGKFLPobpA[4] and pcDNA5-FRT/TO-RNAi plasmids. Cells were selected using Hygromycin (*Sigma*) and colonies expanded. miRNA expression was induced with 1 µg/ml tetracycline (*Sigma*).

Growth Curves

For each stable cell line, $1x10^6$ cells were plated in 10 cm petri dishes in the presence or absence of 1 µg/ml tetracycline (*Sigma*). At two day intervals the cells were trypsinised before being resuspended in 5ml of media. Cells were then counted using an improved Neubauer haemocytometer and the total number of cells per plate was determined. Following counting, $1x10^6$ cells were returned to the dishes and grown for two days in fresh media. This process was repeated for a total of 14 days

Coprecipitation Experiments

Co-immunoprecipitation assays from transfected 293T extracts were performed as described in IP lysis buffer (50mM HEPES pH 7.5, 100 mM NaCl, 1mM EDTA, 1 mM DTT, 0.5% triton X-100, 10% glycerol) in the presence of 5 μg RNase A when indicated. For co-precipitations in denaturing conditions, 293T cells transfected with control (Myc-6His) or Myc-6His-REF2-I and FLAG-UIF were UV-irradiated as indicated on ice with 0.3 J/cm^2. Cells were lysed in 300 μl 4x IP lysis buffer + protease inhibitors before extracts were treated as indicated with 10 μg RNase A. Extracts were then supplemented with 900 μl denaturing solution (8M urea, 0.15% SDS, pH 7.5) and Myc-6His-REF2-I was subjected to purification by Ion Metal Affinity Chromatography (IMAC) using TALON/Co^{2+} beads (*Clontech*). Proteins were eluted from washed beads with 50 μl buffer (50 mM Tris pH 8.0, 100 mM NaCl, 200 mM imidazole) and analysed by Western blotting using α-Myc and α-FLAG monoclonal antibodies.

Pull-down Assays

GST or GB1-tagged proteins expressed in *E. coli* were respectively adsorbed onto 30 μl slurry Glutathione or IgG coated beads (*GE healthcare*) in PBS / 0.1% Tween (Fig. 2C, 4D) or in RB100 buffer (25 mM HEPES pH 7.5, 100 mM KOAc, 10 mM $MgCl_2$, 1 mM DTT, 0.05% Triton X-100, 10% Glycerol) (Fig. 1B,2B,E,5). Pulldowns were performed in previous buffers using 50 μg purified recombinant proteins or 8 μl [^{35}S]-L-methionine-labelled proteins synthesised in rabbit reticulocytes (*Promega*) in the absence or presence of 5 μg RNase A. Proteins were eluted from washed beads in GSH elution buffer (50 mM TRIS, 100 mM NaCl, 40 mM reduced glutathione) or 2x protein loading buffer for GB1 pull downs, before SDS-PAGE analysis, staining

with Coomassie blue and phosphoimaging when indicated. For competition experiment (Fig. 2E), 5 or 50 μg purified GB1-6His-REF2-I were added to GST-NXF1-p15:UIF complexes in 1 ml RB100 buffer.

Immunofluorescence and In Situ Hybridisation

RNA-F.I.S.H. experiments were carried out as described [5]. Cells were treated with actinomycin D (10μg/ml) for two hours prior to fixation to reduce staining from nascent RNA as described [6]. Wholemount *in situ* hybridisation on chick embryos were performed using DIG-labelled (*Roche*) antisense RNA probes generated from chicken cDNA clones chEST420h9 (UIF), chEST65e15 (REF) and chEST96n17 (SF2/ASF)[7] as described [8].

MS2 and RNA Binding Assays

MS2-tethered pre-mRNA reporter export assays were carried out as described [9]. mRNP capture assay was performed as reported [10] except that the TCA precipitation step was removed and proteins were eluted with 50 μl RNAse A buffer. *In vitro* RNA-protein UV cross-linking assays were performed using 5 μg GST, REF2-I-6His or GB1-6His-UIF and a 32 mer [^{32}P]-continuously labelled RNA synthesised from 1 μg *Xba*I-restricted pBluescript-KS with the T7 riboprobe system (*Promega*) in RNA-binding buffer (15 mM HEPES pH 7.9, 100 mM NaCl, 5 mM $MgCl_2$, 0.05% Tween 20, 10% glycerol). Complexes were UV-irradiated on ice before analysis by SDS-PAGE Coomassie staining and phosphoimaging.

Quantitative Analysis of Total and Cytoplasmic mRNA Levels

Trypsin-treated cells were first harvested for Total (3 wells from a 24 well plate) and Cytoplasmic (9 wells from a 24 well plate) preparations. Cytoplasmic fractions were subsequently isolated in 250 μl hypotonic lysis buffer as described [11] and α-SSRP1 Western blotting confirmed the absence of nuclear leakage (Figure S7). Total and cytoplasmic RNA were extracted with 750 μl TRIZOL-LS as indicated by the manufacturer (*Invitrogen*). RNA was treated with amplification grade-DNase I (*Sigma*) and further purified with RNA-phenol (*Stratagene*) and EtOH precipitation. Dried pellets were resuspended in H_2O and 2 μg RNA (quantified by spectrophotometry at OD_{260}) were used for cDNA synthesis using poly(dN)$_6$ random priming as described by the manufacturer (Bioscript kit from *Bioline*). 35 μl H_2O were added to 20 μl cDNA reactions and 1 μl diluted

cDNA with 5 ng/μl primers were used in 10 μl quantitative PCRs (*Quantace*) run on a Rotorgene 6000 (*Qiagen*).

RNA Immunoprecipitation Assays (RIP)

The protocol was adapted from [12]. Trypsin-treated cells from 2x 6cm dishes were subjected to 1% formaldehyde cross-link in 5 ml PBS for 10 min at room temperature before reactions were quenched with 0.25M glycine and cells extensively washed with PBS. Cell pellets were lysed in 1 ml lysis buffer (50mM HEPES pH 7.5, 300 mM NaCl, 1mM EDTA, 1 mM DTT, 0.5% Triton X-100, 10% glycerol) containing protease inhibitors (PMSF and Complete from *Roche*) and RNase (*Bioline*) inhibitors. Lysis was completed by sonication and 100 μl lysates were kept as input while 900 μl were subjected to α-UIF, α-ALY 11G5 (*Sigma*) or null (preimmune UIF serum or α-FLAG for ALY RIP) immunoprecipitations with 40 μl protein G-sepharose (*GE healthcare*):antibody beads. Beads were washed 5 times with lysis buffer and complexes were eluted and reversed with 100 μl RIP elution buffer (lysis buffer, 1% SDS, 10 mM DTT, 5 mM EDTA) during 1 h at 70ºC. RNA was extracted with 300 μl TRIZOL LS (*Invitrogen*) and 80 μl chloroform. Supernatants were precipitated o/n at -20ºC. Dried pellets were treated with amplification grade-DNase I before STOP solution was added (*Sigma*). cDNA synthesis was achieved using poly(dN)$_6$ priming as described by the manufacturer (Bioscript from *Bioline*). 80 μl H_2O were added to 20 μl cDNA reactions and 1 μl diluted cDNA with 5 ng/μl primers were used in 10 μl quantitative PCRs (*Quantace*) run on a Rotorgene 6000 (*Qiagen*). No signal was seen when reverse transcriptase was omitted from reactions (Figure S8).

Primer Pairs Used for Quantitative RT-PCR

Primers were designed using Primer3plus and NCBI programs. Sequences (5'-3'): TBX18 (F: TTTGTCTGGCTCCTCTTGCT, R: ATGGACATGTTCACGGAGGT), ELYS (F: TGATGTCCTTGGTGATGGTG, R: GAGGGCTGTCAGAAATTGGA), GAPDH (F: GAAGGTGAAGGTCGGAGTC, R: GAAGATGGTGATGGGATTTC), EGR-1 (F: CCTCCCTCTCTACTGGAGTGGAA, R: GAAGAACTTGGACATGGCTGTTTC), CALML3 (F: GGCCTTCTCCCTGTTTGAC, R: TCCGTGTCCTTCATCTTCCT), JUN (F: GAACTGCACAGCCAGAACAC, R: TGGGTTGAAGTTGCTGAGG), IER5 (F:

TGGCTAACCTCATCAGCATC, R: GGGTTCATGTCTCTCAGCAC), UIF (F: AGCAGTGCAATGCCCAGTAA, R: ACCGCTCATTCAACGTCATC), REF (F: GCCTGCACAGAGCGTAAACA, R: CTCGCATTATAGGCGTCCAG), UAP56 (F: TCCCCTACTCTTCACCCCTT, R: CTGCTGTCTCCACCTCATCA), DDX39 (F:TCCTCAAGAGAGCACACCAG, R: AGGATGCTCAAAGCCACAGT), NXF1 (F: CCCAAGTTACTACGCCTGGA, R: GCAGGGTTCTGAGGAATGAA), snRNA U6 (F: TGCTCGCTTCGGCAGCACAT, R: AATATGGAACGCTTCACGAA), snRNA U1 (F: ACCTGGCAGGGGAGATACCA, R: GGGGAAAGCGCGAACGCAGT). Run were performed 45 x [10 sec 95ºC / 10 sec 59ºC / 25 sec 72ºC].

Mass Spectrometry

Proteins were identified by mass spectrometry as described previously [13]. Briefly, proteins were subjected to in gel digestion using 200 ng trypsin/band. Peptides were extracted from the gel using acetonitrile, vacuum dried and resuspended in 10 ul 0.1% TFA. The mixture was then separated on a PepMap C-18 reversed phase capillary column (LC Packings, Amsterdam, The Netherlands) and eluted in a 30-minute gradient via a LC Packings Ultimate nanoLC directly onto an Applied Biosystems QStarXL® electrospray ionisation quadrupole time of flight tandem mass spectrometer (ESI qQ-TOF). Mascot 2.2 was used to search the Swissprot database (downloaded 181108) for protein matches. Matches with score >50 were considered significant.

Table S1. Plasmids Used in this Study

Plasmid name	Description	Tag(s)	Source
pcDNA Myc HisA-UAP56	*UAP56* ORF cloned as BamHI/XhoI PCR fragment into pcDNA Myc HisA	Myc + 6 His (3')	Hautbergue et al., 2008
pcDNA Myc HisA-SSRP1	SSRP1 ORF cloned as HindIII/XhoI PCR fragment into pcDNA Myc HisA	Myc + 6 His (3')	This study
pcDNA Myc HisA-HPR1	*HPR1* ORF cloned as BamHI/XhoI PCR fragment into pcDNA Myc HisA	Myc + 6 His (3') ¶	This study
pcDNA Myc HisA-TEX1	*TEX1* ORF cloned as EcoRI/XhoI PCR fragment into pcDNA Myc HisA	Myc + 6 His (3') ¶	This study
pcDNA Myc HisA-SAP35	*SAP35* ORF cloned as BamHI/SalI PCR fragment into pcDNA Myc HisA	Myc + 6 His (3') ¶	This study
pcDNA Myc HisA-CBP80	*CBP80* ORF cloned as EcoRI/XhoI PCR fragment into pcDNA Myc HisA	Myc + 6 His (3') ¶	This study
pcDNA Myc HisB-REF	*REF2-I* ORF cloned as BamHI/XbaI PCR fragmrnt into pcDNA Myc HisB	Myc + 6 His (3')	Williams et al., 2005
pCINeo-LacZ	Full length LacZ subcloned into pCINEO	None	L. Roaden
pCINeo-MS2-REF	*REF2-I* ORF cloned as XbaI/NotI PCR fragment into pCINeo-MS2	MS2 (5') + Myc (3')	Hargous et al., 2006
pCINeo-MS2-GFP	*GFP* ORF cloned as XbaI/NotI PCR fragment into pCINeo-MS2	MS2 (5') + Myc (3')	This study
pCINeo-MS2-UIF	*UIF* ORF cloned as XbaI/NotI PCR fragment into pCINeo-MS2	MS2 (5') + Myc (3')	This study
peGFP-N1		eGFP (3')	Clontech
peGFP-N1-REF	*REF2-I* ORF cloned as XhoI/BamHI PCR fragment into peGFP-N1	eGFP (3')	This study
peGFP-N1-UIF	*UIF* ORF cloned as NheI/XhoI PCR fragment into peGFP-N1	eGFP (3')	This study
peGFP-N1-E1BAP5	*E1BAP5* ORF cloned as EcoRI/BamHI PCR fragment into peGFP-N1	eGFP (3')	E.M. Aguilar-Martinez
pET9a-p15	*p15* ORF cloned as NdeI/BamHI PCR fragment into pET9a	None	Hautbergue et al., 2008
pET9a-TAP-15	PCR-built operon construct expressing TAP-6His and p15		Hautbergue et al., 2008
pET24b-UAP56	*UAP56 ORF* cloned as BamHI/XhoI PCR fragment into pET24b	T7 (5') + 6 His (3')	This study
pET24b-GB1	*GB1+ 6 His* sequence cloned as AseI/NdeI PCR fragment into pET24b	GB1 (5') + T7 + 12 His	Hautbergue et al., 2008
pET24b-GB1-UIF	*UIF* ORF cloned as NdeI/XhoI PCR fragment into pET24b-GB1	GB1 + 6 His (5')	This study
pET24b-GB1-REF	*REF2-I* ORF cloned as NdeI/XhoI PCR fragment into pET24b-GB1	GB1 + 6 His (5') + 6His (3')	This study
pET24b-REF	*REF2-I* ORF cloned as BamHI/XhoI PCR fragment into pET24b	T7 (5') + 6 His (3')	Hautbergue et al., 2008
pET24b-SSRP1	*SSRP1* ORF cloned as NdeI/XhoI PCR fragment into pET24b	6 His (3')	This study
pFlag	p3X-FLAG-Myc CMV26	3x FLAG (5') + Myc (3')	Sigma
pFlag-UIF	*UIF* ORF cloned as NotI/XbaI PCR fragment into pFlag	3x FLAG (5') + Myc (3')	This study
pFlag-REF	*REF2-I* ORF cloned as EcoRI/XbaI PCR fragment into pFlag	3x FLAG (5') + Myc (3') ¶	Hautbergue et al., 2008
pFlag-REF (16-218)	*ref2-I* 46-654 cloned as EcoRI/XbaI PCR fragment into pFlag	3x FLAG (5') + Myc (3') ¶	This study
pFlag-REF (1-198)	*ref2-I* 1-594 cloned as EcoRI/XbaI PCR fragment into pFlag	3x FLAG (5') + Myc (3') ¶	This study
pFlag-REF (16-198)	*ref2-I* 46-594 cloned as EcoRI/XbaI PCR fragment into pFlag	3x FLAG (5') + Myc (3') ¶	This study
pGEX6P1		GST (5')	GE Healthcare
pGEX6P1-REF	*REF2-I* ORF cloned as BamHI/XhoI PCR fragment into pGEX6P1	GST (5')	Hautbergue et al., 2008
pGEX6P1-REF (1-15)	*ref2-I* 1-45 cloned as BamHI/XhoI annealed oligos into pGEX6P1	GST (5')	This study
pGEX-NXF1/TAP		GST (5')	Williams et al., 2005
pIRES-NXF1-13Myc	13Myc tagged *NXF1/TAP* cloned in pIRES-NEO backbone	13 Myc	Hautbergue et al., 2008
pLUCSALRRE6MS2	6 MS2 operators cloned as XhoI fragment into SalI site of pLUCSALRRE	Myc (3')	Williams et al., 2005

Plasmids built to generate stable and inducible RNAi cell lines:

pcDNA6.2-GW/EmGFP-miRNA	Linear plasmid	Invitrogen
pcDNA/EmGFP-single RNAi cassette	Various RNAi cassettes were ligated with pcDNA6.2-GW/EmGFP-miRNA	This study
pcDNA/EmGFP-doubleRNAi cassette	RNAi cassette subcloned as BamHI/XhoI fragment into pcDNA6.2-GW/EmGFP-single RNAi BglII/XhoI	This study
pcDNA5FRT/TO-His		Addgene
pcDNA5FRT/TO-RNAi control	RNAi cassette cloned as HindIII/NotI PCR fragment into pcDNA5FRT/TO	This study
pcDNA5FRT/TO-UAP56i	*UAP56* RNAi cassette #2 cloned as HindIII/NotI PCR fragment into pcDNA5FRT/TO	This study
pcDNA5FRT/TO-DDX39i	*DDX39* RNAi cassette #3 cloned as HindIII/NotI PCR fragment into pcDNA5FRT/TO	This study
pcDNA5FRT/TO-UAP56/DDX39i	*UAP56/DDX39* RNAi cassette cloned as HindIII/NotI PCR fragment into pcDNA5FRT/TO	This study
pcDNA5FRT/TO-UIFi	*UIF* RNAi cassette #5 cloned as HindIII/NotI PCR fragment into pcDNA5FRT/TO	This study
pcDNA5FRT/TO-REFi	*REF* RNAi cassette #2 cloned as HindIII/NotI PCR fragment into pcDNA5FRT/TO	This study
pcDNA5FRT/TO-UIF/REFi	*UIF*/*REF* RNAi cassette cloned as HindIII/NotI PCR fragment into pcDNA5FRT/TO	This study
pcDNA5FRT/TO-SSRP1i #2	*SSRP1* RNAi cassette #2 cloned as HindIII/NotI PCR fragment into pcDNA5FRT/TO	This study
pcDNA5FRT/TO-SSRP1i #5	*SSRP1* RNAi cassette #5 cloned as HindIII/NotI PCR fragment into pcDNA5FRT/TO	This study
pcDNA5FRT/TO-SSRP1 #2#5	*SSRP1* RNAi cassette #2, #5 cloned as HindIII/NotI PCR fragment into pcDNA5FRT/TO	This study

¶ NLS sequence (5'-CCA AAA AAG AAG AGA AAG GTC GAA) inserted between the FLAG tag and the beginning of the open reading frames of pFlag plasmids or at the beginning of the open reading frames for the pcDNA Myc His A constructs and cloned within the 5' oligonucleotide used in the PCR recations.

References:

1. Hargous, Y., Hautbergue, G. M., Tintaru, A. M., Skrisovska, L., Golovanov, A. P., Stevenin, J., Lian, L. Y., Wilson, S. A. & Allain, F. H. (2006) *Embo J.* 25, 5126-37.
2. Williams, B. J., Boyne, J. R., Goodwin, D. J., Roaden, L., Hautbergue, G. M., Wilson, S. A. & Whitehouse, A. (2005) *Biochem J* 387, 295-308.
3. Hautbergue, G.M., Hung, M.-L., Golovanov, A.P., Lian L.-Y., Wilson, S.A. (2008) *Proc Natl Acad Sci U S A.* 13, 5154-9.

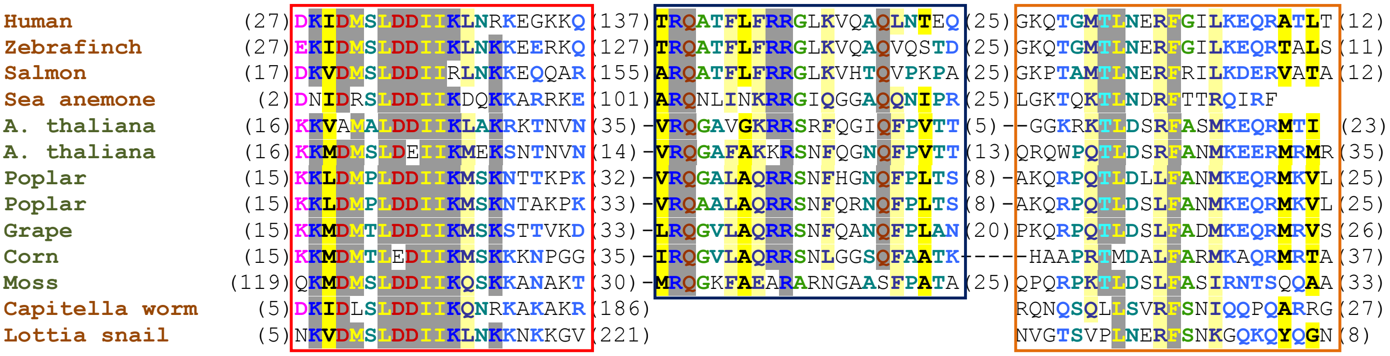

Human (27)DKIDMSLDDIIKLNRKEGKKQ(137)TRQATFLFRRGLKVQAQLNTEQ(25)GKQTGMTLNERFGILKEQRATLT(12)
Zebrafinch (27)EKIDMSLDDIIKLNKKEERKQ(127)TRQATFLFRRGLKVQAQVQSTD(25)GKQTGMTLNERFGILKEQRTALS(11)
Salmon (17)DKVDMSLDDIIRLNKKEQQAR(155)ARQATFLFRRGLKVHTQVPKPA(25)GKPTAMTLNERFRILKDERVATA(12)
Sea anemone (2)DNIDRSLDDIIKDQKKARRKE(101)ARQNLINKRRGIQGGAQQNIPR(25)LGKTQKTLNDRFTTRQIRF
A. thaliana (16)KKVAMALDDIIKLAKRKTNVN(35)-VRQGAVGKRRSRFQGIQFPVTT(5)--GGKRKTLDSRFASMKEQRMTI (23)
A. thaliana (16)KKMDMSLDEIIKMEKSNTNVN(14)-VRQGAFAKKRSNFQGNQFPVTT(13)QRQWPQTLDSRFANMKEERMRMR(35)
Poplar (15)KKLDMPLDDIIKMSKNTTKPK(32)-VRQGALAQRRSNFHGNQFPLTS(8)-AKQRPQTLDLLFANMKEQRMKVL(25)
Poplar (15)KKLDMPLDDIIKMSKNTAKPK(33)-VRQAALAQRRSNFQRNQFPLTS(8)-AKQRPQTLDSLFANMKEQRMKVL(25)
Grape (15)KKMDMTLDDIIKMSKSTTVKD(33)-LRQGVLAQRRSNFQANQFPLAN(20)PKQRPQTLDSLFADMKEQRMRVS(26)
Corn (15)KKMDMTLEDIIKMSKKKNPGG(35)-IRQGVLAQRRSNLGGSQFAATK-----HAAPRTMDALFARMKAQRMRTA(37)
Moss (119)QKMDMSLDDIIKQSKKANAKT(30)-MRQGKFAEARARNGAASFPATA(25)QPQRPKTLDSLFASIRNTSQQAA(33)
Capitella worm (5)DKIDLSLDDIIKQNRKAKAKR(186) RQNQSQLLSVRFSNIQQPQARRG(27)
Lottia snail (5)NKVDMSLDDIIKLNKKNKKGV(221) NVGTSVPLNERFSNKGQKQYQGN(8)

Figure S1. Multiple Sequence Alignment of Animal and Plant UIF-like Sequences

Three regions of high sequence similarity were highlighted by BLOCKMAKER: sequences boxed in red represent the REF-N motif, those boxed in blue and in brown are alignment blocks present in UIF-like sequences, but not in other REF-N-containing proteins. Numbers in parentheses represent amino acids that have been excised from the alignment. Species and GenInfo accession numbers (in parentheses): in order, *Homo sapiens* (147643840); *Taeniopygia guttata* (224060619); *Nematostella vectensis* (156357638); *Arabidopsis thaliana* (15236662); *Arabidopsis thaliana* (15236990); *Populus trichocarpa* (224058254); *Populus trichocarpa* (224072204);*Vitis vinifera* (225426326); *Zea mays* (212722224); *Physcomitrella patens* (168015325); *Capitella* sp. I (161210875); and, *Lottia gigantea* (Lotgi1 assembly; scaffold 23 bases 2114358-2120932).

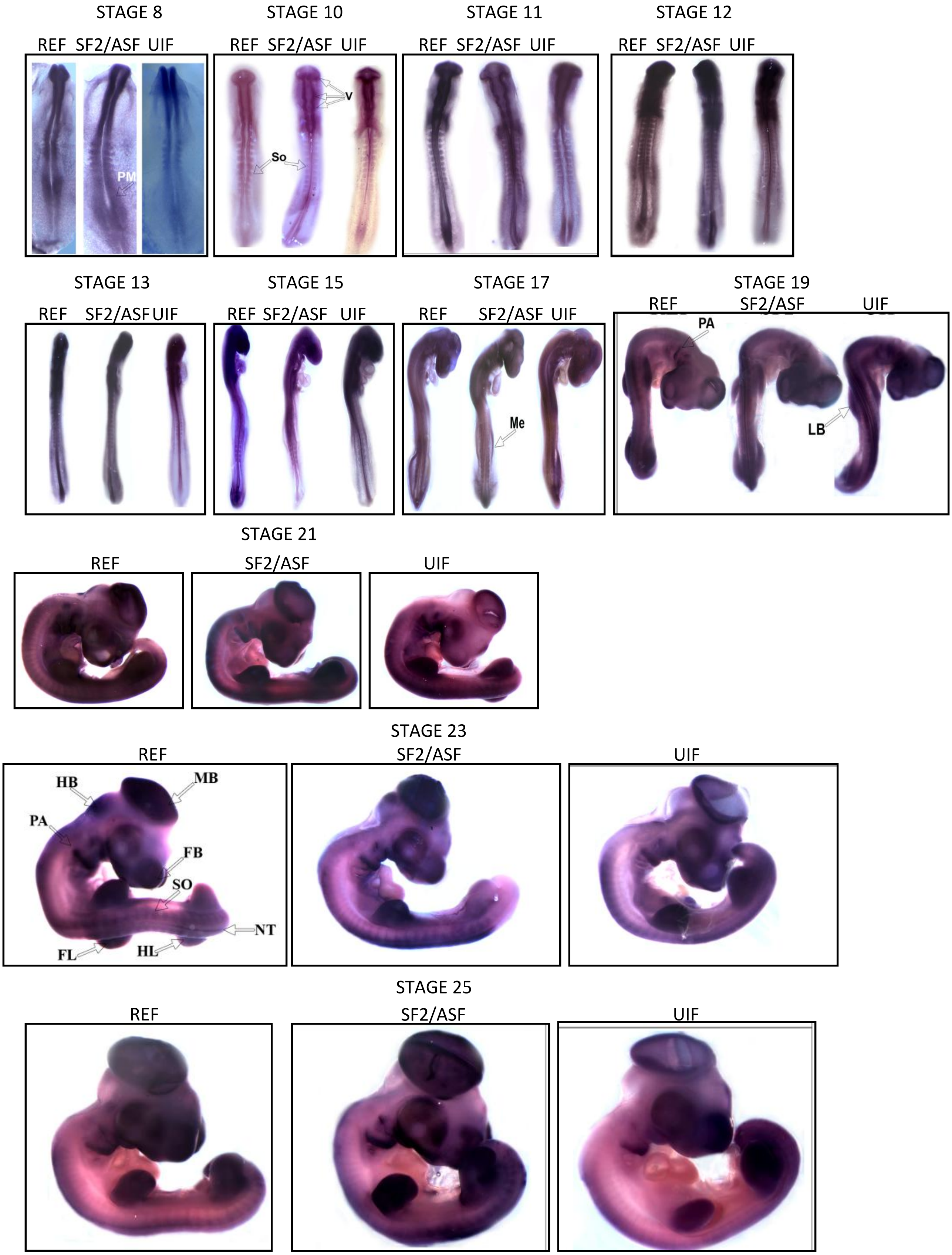
STAGE 8
REF SF2/ASF UIF
PM
STAGE 10
REF SF2/ASF UIF
V
So
STAGE 11
REF SF2/ASF UIF
STAGE 12
REF SF2/ASF UIF
STAGE 13
REF SF2/ASF UIF
STAGE 15
REF SF2/ASF UIF
STAGE 17
REF SF2/ASF UIF
Me
STAGE 19
REF SF2/ASF UIF
PA
LB
STAGE 21
REF
SF2/ASF
UIF
STAGE 23
REF
SF2/ASF
UIF
HB
MB
PA
FB
SO
NT
FL
HL
STAGE 25
REF
SF2/ASF
UIF

Figure S2. Expression of REF, SF2/ASF, and UIF during Development of the Chick Embryo

In situ hybridisation with the indicated probes using chick embryos at different stages of development. PM, paraxial mesoderm; V, the 3 brain vesicles; SO, somites; PA, fifth pouch of the pharyngeal arches; LB, limb bud; HB, hindbrain; FB, forebrain, NT, neural tube; HL, hindlimb; FL, forelimb.

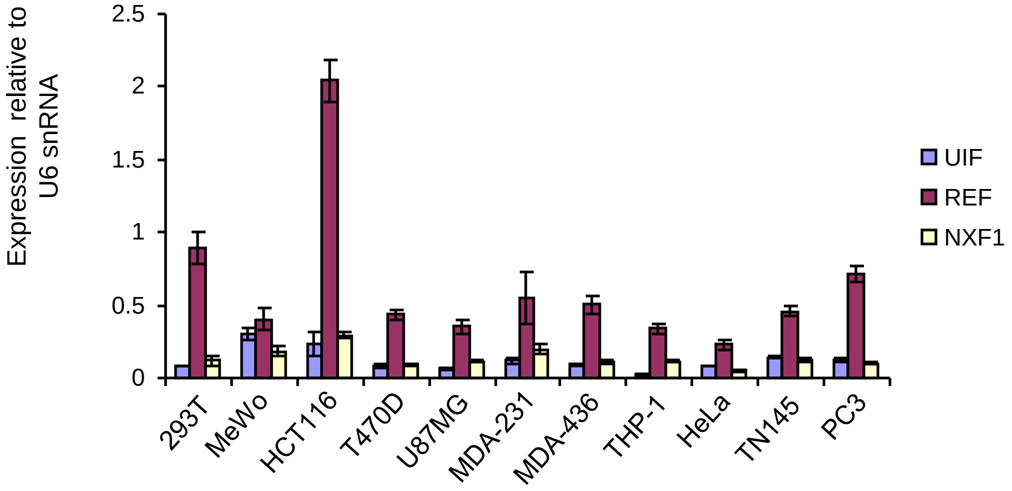
Expression relative to U6 snRNA
2.5
2
1.5
1
0.5
0
293T
MeWo
HCT116
T470D
U87MG
MDA-231
MDA-436
THP-1
HeLa
TN145
PC3
UIF
REF
NXF1

Figure S3. Expression of UIF, REF, and NXF1 in Mammalian Cell Lines

Quantitative RT-PCR analysis was used to assess the levels of each gene relative to the U6 snRNA which was set to a value of 1. Error bars represent the standard error of the mean from 3 experiments

A

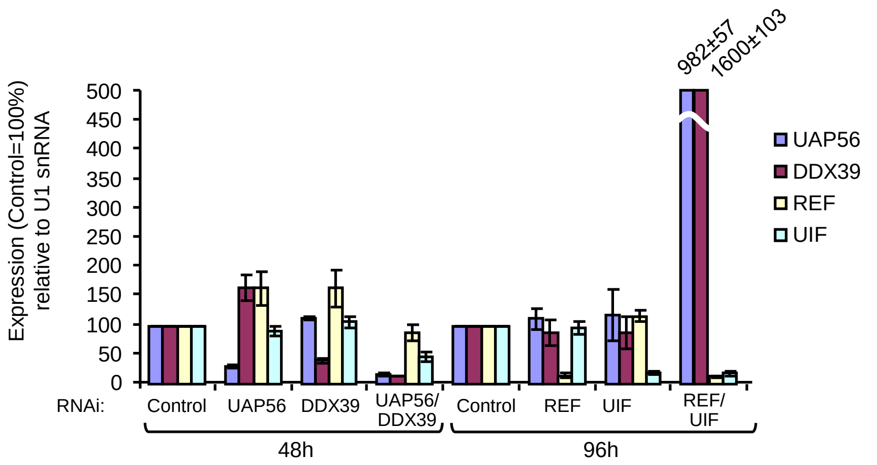
982±57
1600±103
Expression (Control=100%) relative to U1 snRNA
500
450
400
350
300
250
200
150
100
50
0
UAP56
DDX39
REF
UIF
RNAi:
Control
UAP56
DDX39
UAP56/ DDX39
Control
REF
UIF
REF/ UIF
48h
96h

B

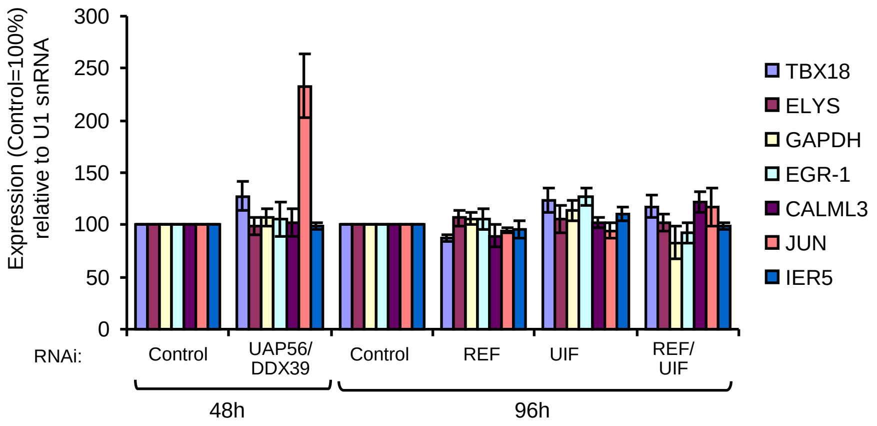
Expression (Control=100%) relative to U1 snRNA
300
250
200
150
100
50
0
TBX18
ELYS
GAPDH
EGR-1
CALML3
JUN
IER5
RNAi:
Control
UAP56/ DDX39
Control
REF
UIF
REF/ UIF
48h
96h

Figure S4. Expression of Various Genes in Several Induced-RNAi Human Cell Lines

Quantitative RT-PCR analysis was used on total RNA to assess the levels of each gene relative to the U1 snRNA.

(A) Efficiency of RNAi was controlled for each RNAi stable cell lines. Error bars represent s.e.m. from 3 experiments. A large increase in UAP56 and DDX39 mRNA levels are seen in the double knockdown of REF/UIF which probably represents the cells attempts rescue mRNA export with higher levels of UAP56/DDX39 mRNA.

(B) Expression of other unrelated genes in the RNAi stable cell lines. Error bars represent s.e.m. from ≥3 experiments.

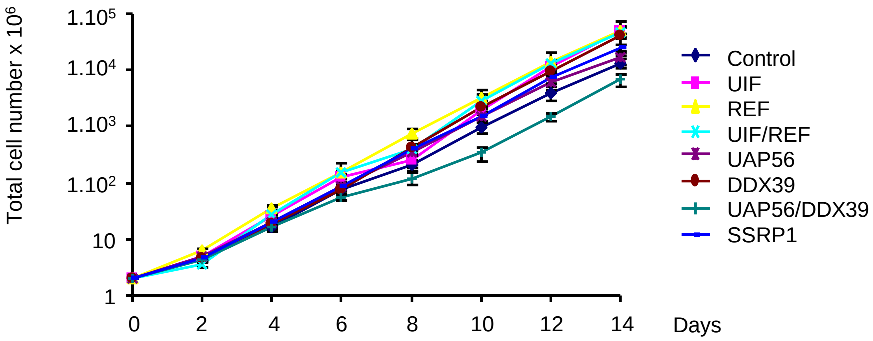

Total cell number x 10^6
1.10⁵
1.10⁴
1.10³
1.10²
10
1
0
2
4
6
8
10
12
14
Days
Control
UIF
REF
UIF/REF
UAP56
DDX39
UAP56/DDX39
SSRP1

Figure S5. Growth of Stable Cell Lines under Noninducing Conditions Harbouring Inducible miRNAs Targeting the Indicated Genes

To minimise any leaky expression of the miRNAs, cells were grown in serum guaranteed free from tetracycline (Clontech). Error bars represent standard deviation from 3 technical replicates.

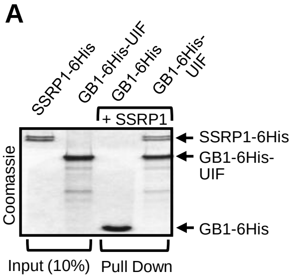
A
SSRP1-6His
GB1-6His-UIF
GB1-6His
GB1-6His-UIF
+ SSRP1
Coomassie
SSRP1-6His
GB1-6His-UIF
GB1-6His
Input (10%)
Pull Down

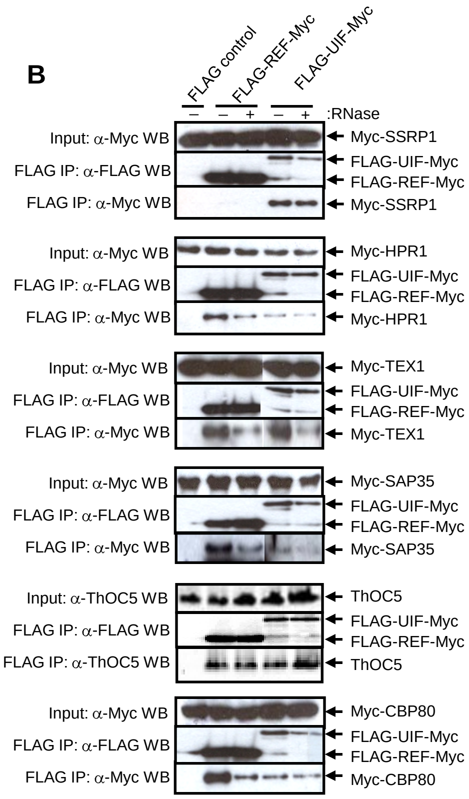
B
FLAG control
FLAG-REF-Myc
FLAG-UIF-Myc
– – + – + :RNase
Input: α-Myc WB
FLAG IP: α-FLAG WB
FLAG IP: α-Myc WB
Myc-SSRP1
FLAG-UIF-Myc
FLAG-REF-Myc
Myc-SSRP1
Input: α-Myc WB
FLAG IP: α-FLAG WB
FLAG IP: α-Myc WB
Myc-HPR1
FLAG-UIF-Myc
FLAG-REF-Myc
Myc-HPR1
Input: α-Myc WB
FLAG IP: α-FLAG WB
FLAG IP: α-Myc WB
Myc-TEX1
FLAG-UIF-Myc
FLAG-REF-Myc
Myc-TEX1
Input: α-Myc WB
FLAG IP: α-FLAG WB
FLAG IP: α-Myc WB
Myc-SAP35
FLAG-UIF-Myc
FLAG-REF-Myc
Myc-SAP35
Input: α-ThOC5 WB
FLAG IP: α-FLAG WB
FLAG IP: α-ThOC5 WB
ThOC5
FLAG-UIF-Myc
FLAG-REF-Myc
ThOC5
Input: α-Myc WB
FLAG IP: α-FLAG WB
FLAG IP: α-Myc WB
Myc-CBP80
FLAG-UIF-Myc
FLAG-REF-Myc
Myc-CBP80

Figure S6. **UIF Interacts with SSRP1, TREX, and CBP80**

(A) Pulldown assays on IgG sepharose using GB1-6His or GB-1-6HIS-UIF together with SSRP1-6His. Assays were carried out in the presence of RNAse. Coomassie stained gel.

(B) Co-IP assays using the indicated FLAG-tagged and Myc tagged cDNAs. The FLAG antibody was used for immunoprecipitation and proteins were detected with both FLAG and Myc antibodies. ThOC5 was detected using an antibody to the endogenous protein.

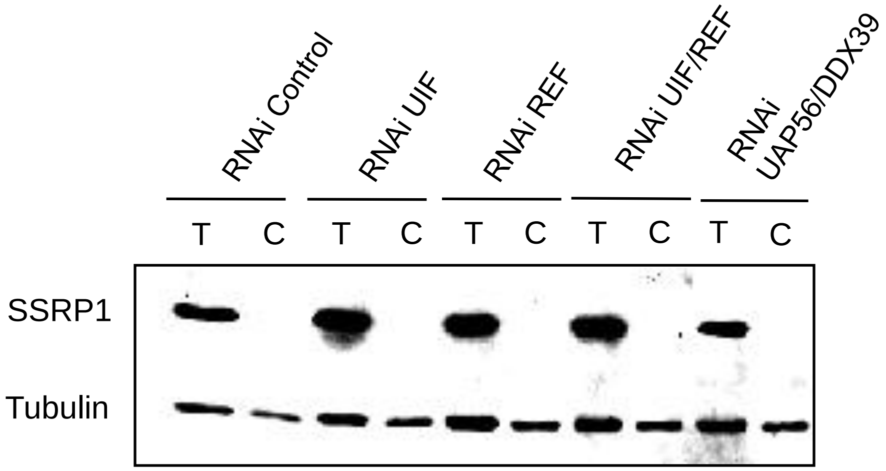
RNAi Control
RNAi UIF
RNAi REF
RNAi UIF/REF
RNAi
UAP56/DDX39
T
C
T
C
T
C
T
C
T
C
SSRP1
Tubulin

Figure S7. Western Blot Analysis

Western blot analysis of total (T) and cytoplasmic (C) extracts from various induced-RNAi stable cell lines using antibodies directed against SSRP1 (nuclear protein) and Tubulin (cytoplasmic protein). No nuclear leakage was observed in the cytoplasmic fractions.

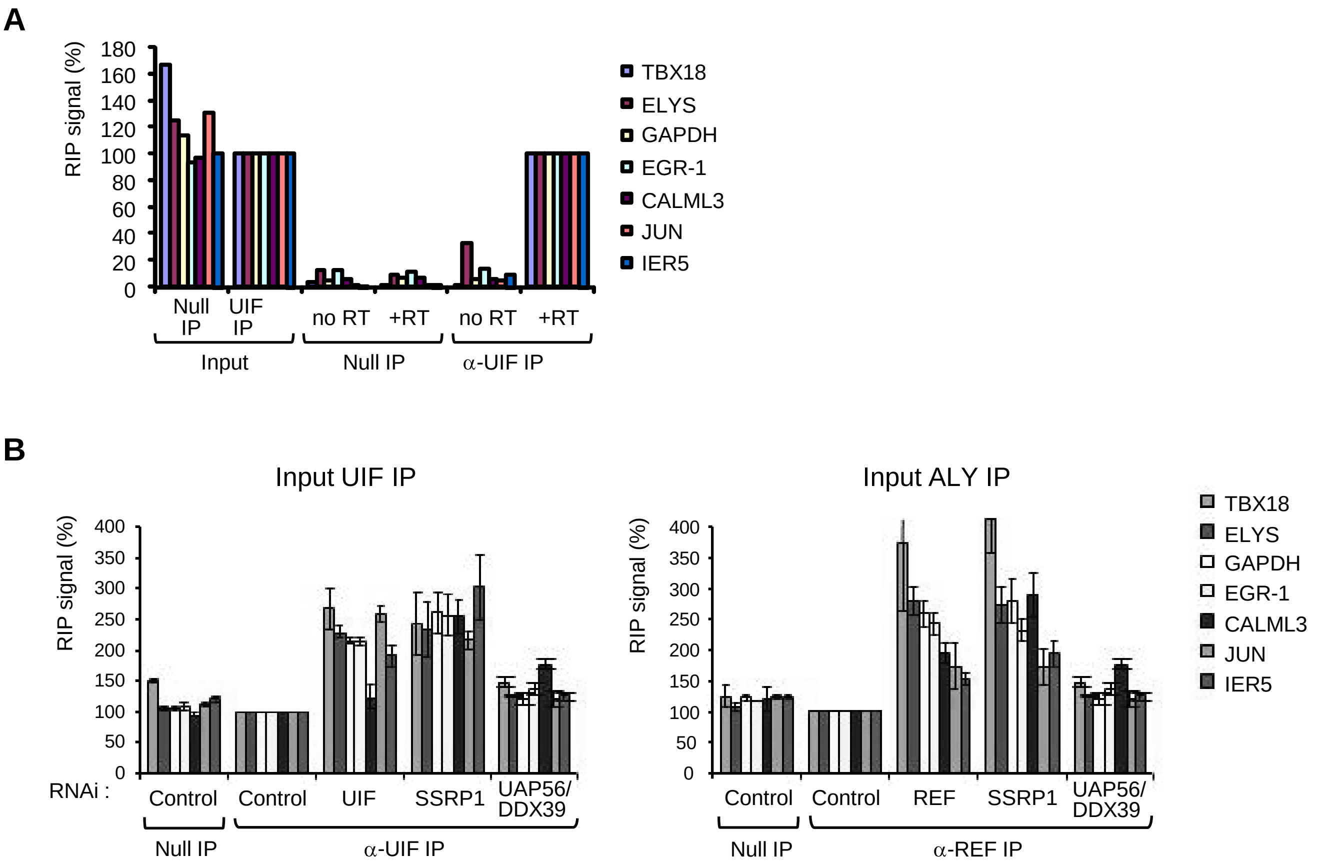
A
RIP signal (%)
180
160
140
120
100
80
60
40
20
0
Null IP
UIF IP
no RT
+RT
no RT
+RT
Input
Null IP
α-UIF IP
TBX18
ELYS
GAPDH
EGR-1
CALML3
JUN
IER5
B
Input UIF IP
Input ALY IP
RIP signal (%)
400
350
300
250
200
150
100
50
0
RNAi :
Control
Control
UIF
SSRP1
UAP56/ DDX39
Null IP
α-UIF IP
Control
Control
REF
SSRP1
UAP56/ DDX39
Null IP
α-REF IP
TBX18
ELYS
GAPDH
EGR-1
CALML3
JUN
IER5

Figure S8. Quantitative RT-PCR Controls

(A) Specificity of qRT-PCR amplifications used to measure the amount of immunoprecipitated RNA in the RIP assays (Fig. 4) was controlled in absence and presence of reverse transcriptase (RT) during synthesis of the cDNA. Input for UIF IP and UIF IP + RT were set up to 100% for each gene.

(B) Aliquots of input RNA for each IP performed in the RIP assays (Fig. 4) were analysed by quantitative RT-PCR to check that the decrease in the amounts of RNA immunoprecipitated was not due to a lower amount of RNA in the input. Error bars represent standard error of the mean from ≥3 experiments.

Supplemental References

1. Henikoff, S., Henikoff, J.G., Alford, W.J., and Pietrokovski, S. (1995). Automated construction and graphical presentation of protein blocks from unaligned sequences. Gene *163*, GC17-26.
2. Goodstadt, L., and Ponting, C.P. (2001). CHROMA: consensus-based colouring of multiple alignments for publication. Bioinformatics *17*, 845-846.
3. Hageman, J., and Kampinga, H.H. (2009). Computational analysis of the human HSPH/HSPA/DNAJ family and cloning of a human HSPH/HSPA/DNAJ expression library. Cell Stress Chaperones *14*, 1-21.
4. Raymond, C.S., and Soriano, P. (2007). High-efficiency FLP and PhiC31 site-specific recombination in mammalian cells. PLoS ONE *2*, e162.
5. Williams, B.J., Boyne, J.R., Goodwin, D.J., Roaden, L., Hautbergue, G.M., Wilson, S.A., and Whitehouse, A. (2005). The prototype gamma-2 herpesvirus nucleocytoplasmic shuttling protein, ORF 57, transports viral RNA via the cellular mRNA export pathway. Biochem J *387*, 295-308.
6. Yoh, S.M., Cho, H., Pickle, L., Evans, R.M., and Jones, K.A. (2007). The Spt6 SH2 domain binds Ser2-P RNAPII to direct Iws1-dependent mRNA splicing and export. Genes Dev *21*, 160-174.
7. Boardman, P.E., Sanz-Ezquerro, J., Overton, I.M., Burt, D.W., Bosch, E., Fong, W.T., Tickle, C., Brown, W.R., Wilson, S.A., and Hubbard, S.J. (2002). A comprehensive collection of chicken cDNAs. Curr Biol *12*, 1965-1969.
8. Ohyama, K., Ellis, P., Kimura, S., and Placzek, M. (2005). Directed differentiation of neural cells to hypothalamic dopaminergic neurons. Development *132*, 5185-5197.
9. Hargous, Y., Hautbergue, G.M., Tintaru, A.M., Skrisovska, L., Golovanov, A.P., Stevenin, J., Lian, L.Y., Wilson, S.A., and Allain, F.H. (2006). Molecular basis of RNA recognition and TAP binding by the SR proteins SRp20 and 9G8. Embo J *25*, 5126-5137.
10. Sanford, J.R., Ellis, J.D., Cazalla, D., and Caceres, J.F. (2005). Reversible phosphorylation differentially affects nuclear and cytoplasmic functions of splicing factor 2/alternative splicing factor. Proc Natl Acad Sci U S A *102*, 15042-15047.
11. Herold, A., Teixeira, L., and Izaurralde, E. (2003). Genome-wide analysis of nuclear mRNA export pathways in Drosophila. Embo J *22*, 2472-2483.
12. Niranjanakumari, S., Lasda, E., Brazas, R., and Garcia-Blanco, M.A. (2002). Reversible cross-linking combined with immunoprecipitation to study RNA-protein interactions in vivo. Methods *26*, 182-190.
13. Snijders, A.P., Walther, J., Peter, S., Kinnman, I., de Vos, M.G., van de Werken, H.J., Brouns, S.J., van der Oost, J., and Wright, P.C. (2006). Reconstruction of central carbon metabolism in Sulfolobus solfataricus using a two-dimensional gel electrophoresis map, stable isotope labelling and DNA microarray analysis. Proteomics *6*, 1518-1529.
